# Diagnostic and Clinical Implications of High Spleen‐To‐Liver Stiffness Ratio in MASH—A Prospective, Comparative Study

**DOI:** 10.1111/liv.70261

**Published:** 2025-08-30

**Authors:** Christian Sebesta, Mathias Jachs, Lukas Hartl, Michael Schwarz, Lorenz Balcar, Benedikt S. Hofer, Nina Dominik, Georg Kramer, Bernhard Scheiner, Albert F. Stättermayer, Benedikt Simbrunner, Till Schöchtner, Friedrich Haimberger, Nicolas Balutsch, Michael Trauner, Mattias Mandorfer, Thomas Reiberger, David J. M. Bauer

**Affiliations:** ^1^ Division of Gastroenterology and Hepatology, Department of Medicine III Medical University of Vienna Vienna Austria; ^2^ Vienna Hepatic Hemodynamic Lab, Division of Gastroenterology and Hepatology, Department of Medicine III Medical University of Vienna Vienna Austria; ^3^ LBG Clinical Research Group MOTION Medical University of Vienna Vienna Austria; ^4^ Christian‐Doppler Laboratory for Portal Hypertension and Liver Fibrosis Medical University of Vienna Vienna Austria; ^5^ Department of Internal Medicine IV Klinik Ottakring Vienna Austria

**Keywords:** AIXPLORE, cirrhosis, liver stiffness, spleen stiffness, SSM/LSM‐ ratio

## Abstract

**Background:**

Liver stiffness measurement (LSM) and spleen stiffness measurement (SSM) represent non‐invasive surrogates of portal hypertension (PH) that both correlate with hepatic venous pressure gradient (HVPG). SSM may overcome limitations of HVPG and LSM in detecting presinusoidal PH components. We investigated the SSM/LSM ratio as a PH surrogate and its relationship to HVPG and spleen diameter across different liver disease aetiologies.

**Methods:**

399 consecutive patients with compensated liver disease undergoing same‐day measurement of HVPG, LSM and SSM were prospectively included.

**Results:**

While patients with alcohol‐related liver disease (ALD; *n* = 200) showed higher LSM (median: 45.5 kPa) and HVPG (15.0 mmHg) than patients with metabolic dysfunction–associated steatohepatitis (MASH; *n* = 49; LSM: 20.9 kPa; HVPG: 12.0 mmHg), their SSM (median: 58.8 vs. 52.8 kPa; *p* = 0.868) was not significantly different. Consequently, the SSM/LSM ratio was higher in MASH (1.66) vs. ALD (1.28), but highest in patients with non‐cirrhotic PH (3.19). When adjusting for HVPG, SSM and spleen diameter remained significantly higher in MASH than in ALD at any given HVPG.

**Conclusions:**

This study demonstrates that SSM/LSM ratios vary across different liver disease aetiologies. Since MASH patients—after adjusting for liver disease severity—show higher SSM/LSM ratios and larger spleen diameters than ALD, our results support the concept of a presinusoidal component of PH in MASH patients.

**Summary**

- We used non‐invasive imaging to study how liver and spleen stiffness relate to pressure in the liver's blood vessels (known as PH) across different types of liver disease.
- We found that patients with fatty liver disease (MASH) had relatively higher spleen stiffness compared to liver stiffness than those with ALD, even when the disease severity was similar.
- This suggests that in MASH, increased pressure may start before it reaches the liver, pointing to a different underlying disease mechanism.

**Abbreviations**
AIH: autoimmune liver disease
ALD: alcoholic liver disease
BMI: body mass index
CSPH: clinically significant portal hypertension
HCC: hepatocellular carcinoma
HVPG: hepatic venous pressure gradient
LSM: liver stiffness measurement
LTX: liver transplant
MASH: metabolic dysfunction–associated steatohepatitis
MELD: model of endstage liver disease
N: number
PSVD: portal sinusoidal vascular disease
PVT: portal vein thrombosis
SSI: supersonic shear imaging
SSM: spleen stiffness measurement
SWE: shear wave elastography
TIPS: transjugular intrahepatic portosystemic shunt
US: ultrasound
VCTE: vibration‐controlled transient elastography
vWF: von Willebrand factor

## 1 Introduction

The development of clinically significant portal hypertension (CSPH) as defined by hepatic venous pressure gradient (HVPG) of ≥ 10 mmHg represents a watershed moment in the natural history of patients with advanced chronic liver diseases (ACLD), after which the risk of hepatic decompensation [1, 2, 3] and liver‐related mortality is considerably increased [4, 5].

Portal hypertension (PH) has distinct structural and dynamic components [6] and can be of prehepatic, intrahepatic, and posthepatic origin. Hepatic PH is further classified into presinusoidal and sinusoidal PH, with some ACLD aetiologies such as cholestatic liver diseases, showing a pronounced presinusoidal PH component [7]. While HVPG is the gold standard for assessing sinusoidal PH, it cannot accurately quantify prehepatic and presinusoidal PH [8, 9].

Recent research suggests that metabolic dysfunction–associated steatohepatitis (MASH) may have a presinusoidal PH component, as HVPG measurements in MASH are less accurate in reflecting directly measured portal pressure as compared to other aetiologies [10, 11]. In fact, MASH patients experience hepatic decompensation at lower HVPG than patients with viral hepatitis [12]. The distinct pathomechanism of PH in MASH is not fully understood, but periportal vascular damage and periportal fibrosis are hypothesised to play significant roles [11]. In the course of liver damage, small bile ducts in the portal areas proliferate, which is thought to precede progressive portal fibrosis [13, 14, 15], while portal inflammation is interpreted as a marker of advanced MASH [13, 16]. Direct portal pressure measurement is rarely performed [17] and has notable limitations [18]. Thus, there is a clinical need for accurate non‐invasive methods to assess the severity of PH cumulating from both sinusoidal and presinusoidal components.

LSM and SSM have emerged as promising alternatives. Clinical algorithms based on LSM in combination with platelet count (PLT), accounting for body mass index (BMI), have been developed to assess the risk for CSPH [19, 20]. Recent studies have demonstrated that SSM predicts the development of varices and variceal bleeding with higher accuracy than LSM or PLT alone [21]. Importantly, the addition of SSM to LSM and PLT improves the accuracy for CSPH risk assessment [22, 23, 24].

The fibrosis pattern and pathomechanisms underlying elevated LSM and SSM vary across liver diseases; for example, alcohol‐related liver disease (ALD) primarily causes lobular inflammation, while viral hepatitis C (HCV) leads to portal inflammation [25]. In HCV, LSM decreases after cure, reflecting both reduced inflammation and the fibrosis reversal [21]—but the relative contributions of these to decrease LSM remain unclear [26, 27]. Recent research has revealed that a SSM/LSM ratio > 2 can aid in diagnosing porto‐sinusoidal vascular liver disease (PSVD) that potentially causes (presinusoidal) CSPH in the absence of cirrhosis [28]. Furthermore, the SSM/LSM ratio was suggested to differentiate between PH associated with cystic fibrosis‐associated liver disease or with PSVD [29]. These findings suggest that the SSM/LSM ratio could help determine the full extent of PH in liver diseases by considering a potential presinusoidal PH component, such as in MASH or PSVD, that is not (fully) captured via HVPG measurements.

Thus, we set up a prospective study (AIXPLORE) to assess the correlation of LSM and SSM and SSM/LSM ratios to HVPG and non‐invasive CSPH surrogates in patients across different liver disease aetiologies.

## 2 Patients and Methods

### 2.1 Study Cohort

This prospective study included patients with suspected or diagnosed compensated ACLD who underwent HVPG measurement, SSI‐LSM and SSI‐SSM, and laboratory testing at the Division of Gastroenterology and Hepatology of the Medical University of Vienna, between July 2020 and November 2023.

**FIGURE 1:**
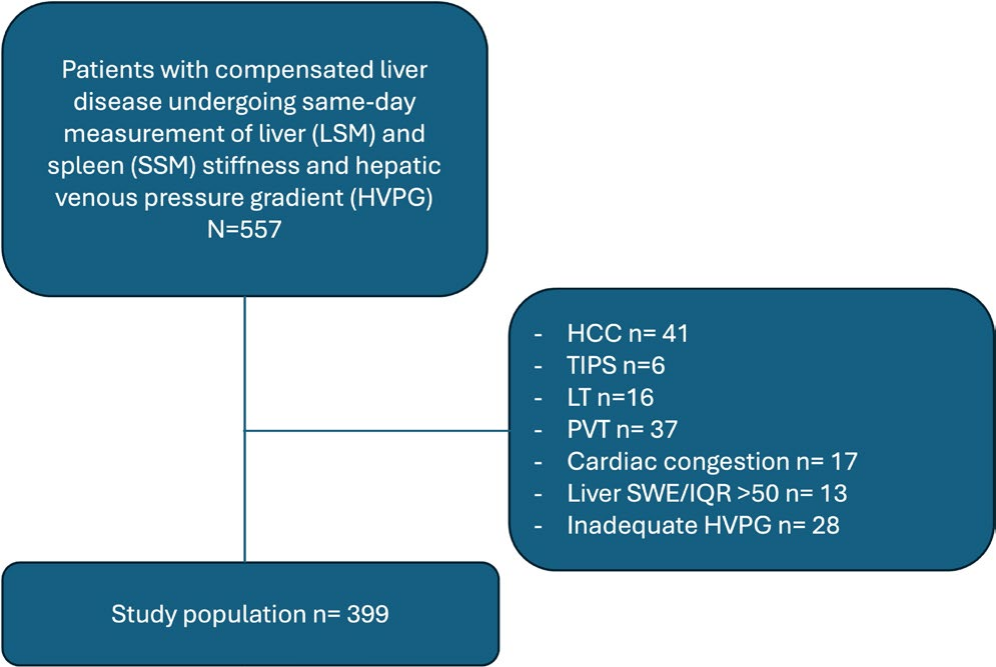
Study population flowchart. Abbreviations: HCC, hepatocellular carcinoma; HVPG, hepatic venous pressure gradient; LSM, liver stiffness measurement; LT, liver transplant; *N*, number; PVT, portal vein thrombosis; SSM, spleen stiffness measurement; SWE, shear wave elastography; TIPS, transjugular intrahepatic portosystemic shunt.

Exclusion criteria comprised hepatocellular carcinoma (HCC), transjugular intrahepatic portosystemic shunt (TIPS), liver transplantation (LT), portal vein thrombosis (PVT) and cardiac congestion failure (Figure 1).

Patients were categorised into the following aetiology groups: MASH, ALD, PSVD, viral, and cholestatic‐related liver disease.

The local ethics committee approved the study protocol (EK‐number: 1544/2019) and informed consent was obtained from all patients. All analyses were conducted in accordance with the 1964 Declaration of Helsinki and its latest amendment.

### 2.2 Elastography

Participants underwent SSI‐2D‐LSM and ‐SSM using the Aixplorer Ultimate ultrasound platform (SSI, Aix‐en‐Provence, France) prior to HVPG measurements. Skilled operators (BAD, BAL, HAL, JAM, MAM, PAR, RET, STA—in alphabetical order), experienced with the Aixplorer platform, performed the elastographies. Operator blindness was ensured due to the timing of diagnostics (elastography preceding HVPG measurements).

To optimise imaging, subjects were asked to lie on their backs, while elevating their right arm and positioning it behind their head, widening the intercostal spaces for improved liver access. The liver tissue was scanned in the mid‐axillary line, employing fanning and transverse movements. Using B‐mode, a measurement area free from blood vessels and other anatomical structures was identified, located at least 2 cm below the organ's capsule. Patients were instructed to pause breathing mid‐exhalation during measurements. Within this area, two 1‐cm regions of interest (ROIs) were selected. This procedure was carried out until at least six separate measurements for both LSM and SSM were obtained for each patient. Splenomegaly was defined as a spleen size exceeding 12 cm in the longest axis.

Vibration‐controlled transient elastography (VCTE)‐LSM via FibroScan (Echosens, Paris, France) was acquired similarly following international recommendations [30, 31]. Patients fasted for a minimum of four hours prior to all measurements. The M‐probe was used initially, with the XL‐probe employed when suggested by the probe selection tool or for patients with a BMI of ≥ 30 kg/m^2^.

### 2.3 Laboratory Parameters

Blood from the participants was drawn and analysed in the ISO‐15189‐certified laboratory of the general hospital in Vienna. Previous diagnoses and clinical parameters were extracted from the hospital's electronic patient records, if not recorded directly.

### 2.4 Statistical Analysis

Chi‐squared test was used to compare frequencies in categorical variables. They are presented as absolute (*N*) and relative frequencies (%). Mean +/− standard deviation (SD) was used for normally distributed continuous variables, while the median and the interquartile range (IQR) were used for non‐normally distributed continuous variables. To compare numeric variables, the Kruskal‐Wallis test was used.

Pearson correlations (Pearson R) were calculated to assess the relationships between 2D‐SWE‐LSM, 2D‐SWE‐SSM, 2D‐SWE‐SSM/LSM ratio, and HVPG. We stratified the strength of the correlation into four categories, “very strong”, “moderate”, “fair” and “poor” [32]. Scatterplots were generated to visualise these correlations. Results with a *p*‐value of ≤ 0.05 were considered statistically significant.

These analyses were performed for the overall group and separately for each liver disease aetiology.

A post hoc power analysis was performed to assess the ability to detect differences in SSM/LSM ratios between MASH and ALD groups based on the observed effect size. Additionally, a multivariable linear regression model was used to evaluate the association between MASH and the SSM/LSM ratio, adjusting for BMI, diabetes mellitus, statin intake, and arterial hypertension.

In order to assess the prognostic meaning of an increased SSM/LSM ratio, we performed a cumulative incidence analysis for decompensation, accounting for HCC, LT, and non‐liver‐related death as competing events. We used Grey's test to compare the cumulative incidence functions between groups of high vs. low SSM/LSM ratio—divided by median SSM/LSM ratio. Statistical analyses were performed using the R programming language and environment for statistical computing (Version 2023.12.1 + 402). The specific R packages utilised are detailed in the Supporting Information.

## 3 Results

### 3.1 Patient Characteristics

Among 557 patients evaluated for this study, 158 were excluded and thus, the final study cohort comprised 399 patients (Figure 1; Table 1). The main aetiology was ALD (*n* = 200; 50.1%) while *n* = 59 patients had viral liver disease, *n* = 49 (12.3%) had MASH and *n* = 18 (4.5%) had PSVD.

**TABLE 1:** Patient characteristics.

	Total population *N* = 399	ALD *N* = 200	MASH *N* = 49	*p* ALD vs. MASH	PSVD *N* = 18
Age (years)	56.0 [48.5–64.0]	57.0 [51.0–63.3]	61.0 [56.0–65.0]	0.0136	51.0 [41.8–55.8]
Male sex (*n*, %)	271 (67.9%)	148 (74.0%)	30 (61.2%)	0.110	11 (61.1%)
BMI	26.0 [22.5–30.0]]	26.0 [22.7–29.9]	32.8 [27.9–36.8]	< 0.001	26.4 [22.7–29.4]
MELD	11.0 [9.0–14.0]	11.0 [10.0–15.3]	9.00 [8.0–11.0]	< 0.001	9.0 [7.0–12.8]
HVPG	14.0 [9.0–18.0]	15.0 [11.0–19.0]	12.0 [8.0–15.0]	< 0.001	5.5 [4.3–7.0]
CSPH	289 (72.4%)	173 (86.5%)	31 (63.3%)	< 0.001	1 (5.6%)
Varices	173 (48.5%)	94 (47.0%)	17 (34.7%)	0.162	9 (52.9%)
2D‐SWE‐LSM (kPa)	33.2 [16.5–59.8]	45.5 [25.1–63.9]	20.9 [15.7–55.0]	0.003	11.2 [7.9–14.4]
2D‐SWE‐SSM (kPa)	53.9 [35.0–69.3]	58.8 [39.7–71.4]	52.8 [34.3–82.6]	0.868	42.6 [33.3–61.9]
2D‐SWE‐SSM/LSM ratio	1.48 [1.0–2.6]	1.28 [0.1–1.8]	1.66 [1.2–2.9]	0.001	3.19 [2.0–5.5]
Adjusted 2D‐SWE‐SSM ratio (by LSM*10)	0.47 [0.2–1.6]	0.29 [0.2–0.7]	0.96 [0.2–2.0]	0.001	2.5 [1.7–6.6]
Platelets (G/L)	110 [77.0–165.0]	110 [79.0–163.0]	127 [92.0–171.0]	0.174	87.0 [53.0–157.0]
AST (U/L)	39.0 [29.0–58.0]	38.0 [28.0–58.0]	43.5 [33.3–58.8]	0.411	32.0 [24.0–41.0]
ALT (U/L)	30.0 [22.0–47.0]	27.0 [20.0–36.0]	43.5 [29.0–67.0]	< 0.001	32.5 [22.3–50.0]
GGT (U/L)	86.0 [40.0–169.0]	107 [56.0–214.0]	114 [62.5–208.0]	0.665	66.0 [29.3–117.0]
vWF > 240% (*n*, %)	210 (57.1%)	117 (58.5%)	23 (46.9%)	0.046	5 (27.8%)
Spleen (cm)	13.3 [11.7–15.5]	13.1 [11.7–15.0]	13.6 [11.5–14.8]	0.758	15.7 [12.0–17.6]
Splenomegaly > 12 cm (*n*, %)	279 (69.9%)	138 (69.0%)	35 (71.4%)	0.875	13 (70.2%)
Spleen Diameter/HVPG ratio	1.02 [0.76–1.53]	0.85 [0.67–1.13]	1.15 [0.83–1.62]	< 0.001	2.94 [2.22–3.80]

Abbreviations: ALD, alcoholic liver disease; BMI, body mass index; CSPH, clinically significant portal hypertension; HVPG, hepatic venous pressure gradient; LSM, liver stiffness measurement; MASH, metabolic dysfunction–associated steatohepatitis; MELD, model of endstage liver disease; *N*, number; PSVD, portal sinusoidal vascular disease; SSI, super sonic imaging; SSM, spleen stiffness measurement; US, ultrasound; VCTE, vibration‐controlled transient elastography; vWF, von Willebrand factor.

ALD patients had a median MELD of 11.0 [IQR: 10.0–15.3] and HVPG of 15.0 [11.0–19.0] mmHg, while MASH had a significantly lower MELD (median: 9; IQR: 8.0–11.0, *p* < 0.001) and HVPG (median: 12.0, IQR: 8.0–15.0, *p* < 0.001).

### 3.2 Correlation of 2D‐SWE‐LSM, 2D‐SWE‐SSM and Their Ratio to HVPG Across the Overall, ALD and MASH Cohorts

**FIGURE 2:**
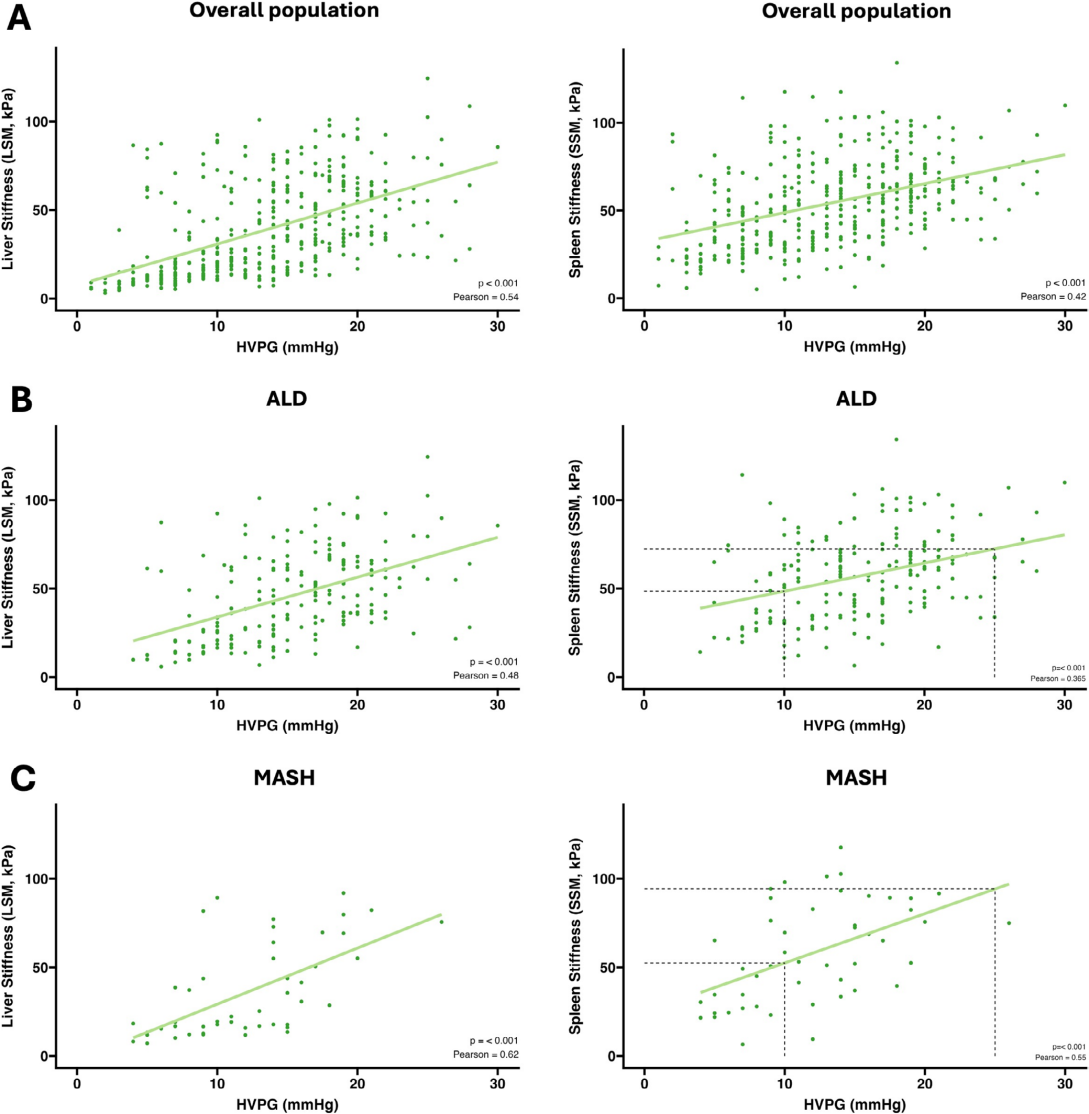
Scatterplots of 2D‐SWE‐Liver (LS) and 2D‐SWE‐spleen stiffness (SS) versus hepatic venous pressure gradient (HVPG). Scatterplots of 2D‐SWE‐LSM (left)/2D‐SWE‐SSM (right) (*y*‐axis) to HVPG (*x*‐axis) in different etiologies (A: Overall, B: ALD, C: MASH), the grey dotted lines indicate the slope of SSM increase within the HVPG range of 10 to 25 mmHg. Abbreviations: ALD, Alcoholic liver disease; HVPG, hepatic venous pressure gradient; LSM, Liver stiffness measurement; MASH, metabolic dysfunction–associated steatohepatitis; SSI, supersonic shear imaging.

The correlations of 2D‐SWE‐LSM and 2D‐SWE‐SSM with HVPG are shown in Figure 2. We found a moderate correlation between 2D‐SWE‐LSM and HVPG in the overall (Pearson *R* = 0.54, *p* < 0.001) as well as the MASH group (Pearson *R* = 0.62, *p* < 0.001), and a fair correlation in the subgroup of patients with ALD (Pearson *R* = 0.48, *p* < 0.001).

Conversely, 2D‐SWE‐SSM showed a fair correlation with HVPG in the overall and ALD cohorts (Overall: Pearson *R* = 0.42, *p* < 0.001; ALD: Pearson *R* = 0.365, *p* < 0.001), while MASH had a moderate correlation (Pearson *R* = 0.55, *p* < 0.001).

The 2D‐SWE‐SSM/LSM ratio exhibited a fair inverse correlation with HVPG across all aetiologies (Pearson *R* = −0.35, *p* < 0.001; Figure S1), in ALD (ALD: Pearson *R* = −0.27, *p* < 0.001) and MASH patients (MASH: Pearson *R* = −0.28, *p* = 0.05).

### 3.3 ALD Had Higher 2D‐SWE‐LSM Than MASH, but 2D‐SWE‐SSM/LSM Was Higher in MASH

Significant differences in 2D‐SWE‐LSM were detected between the aetiologies (*p* = < 0.001). ALD patients exhibited the highest median 2D‐SWE‐LSM of 45.5 [25.1–63.9] kPa, followed by viral liver disease 23.5 [13.8–41.9] and MASH with 20.9 [15.7–55.0] kPa as shown in Table S1.

2D‐SWE‐SSM were not significantly different between ALD and MASH patients: 58.8 [39.7–71.4] vs. 52.8 [34.3–82.6] kPa (*p* = 0.868). Consequently, the 2D‐SWE‐SSM/LSM ratios were significantly higher in MASH (1.66 [1.22–2.94]) than in ALD patients (1.28 [0.963–1.83]; *p* = 0.001). As expected, patients with PSVD—a liver disease causing non‐cirrhotic PH that is not reflected by HVPG (median 5.50 mmHg) and by LSM (median 11.2 kPa), but by SSM (median 42.6 kPa)—showed the highest median SSM/LSM ratio (median 3.19; Table 1). A post hoc analysis confirmed that the statistical power to detect the observed mean difference in the SSM/LSM ratio between the MASH and ALD groups was 88% (*α* = 0.05), corresponding to a medium effect size (Cohen's *d* = 0.51, 95% CI: 0.19–0.84).

**FIGURE 3:**
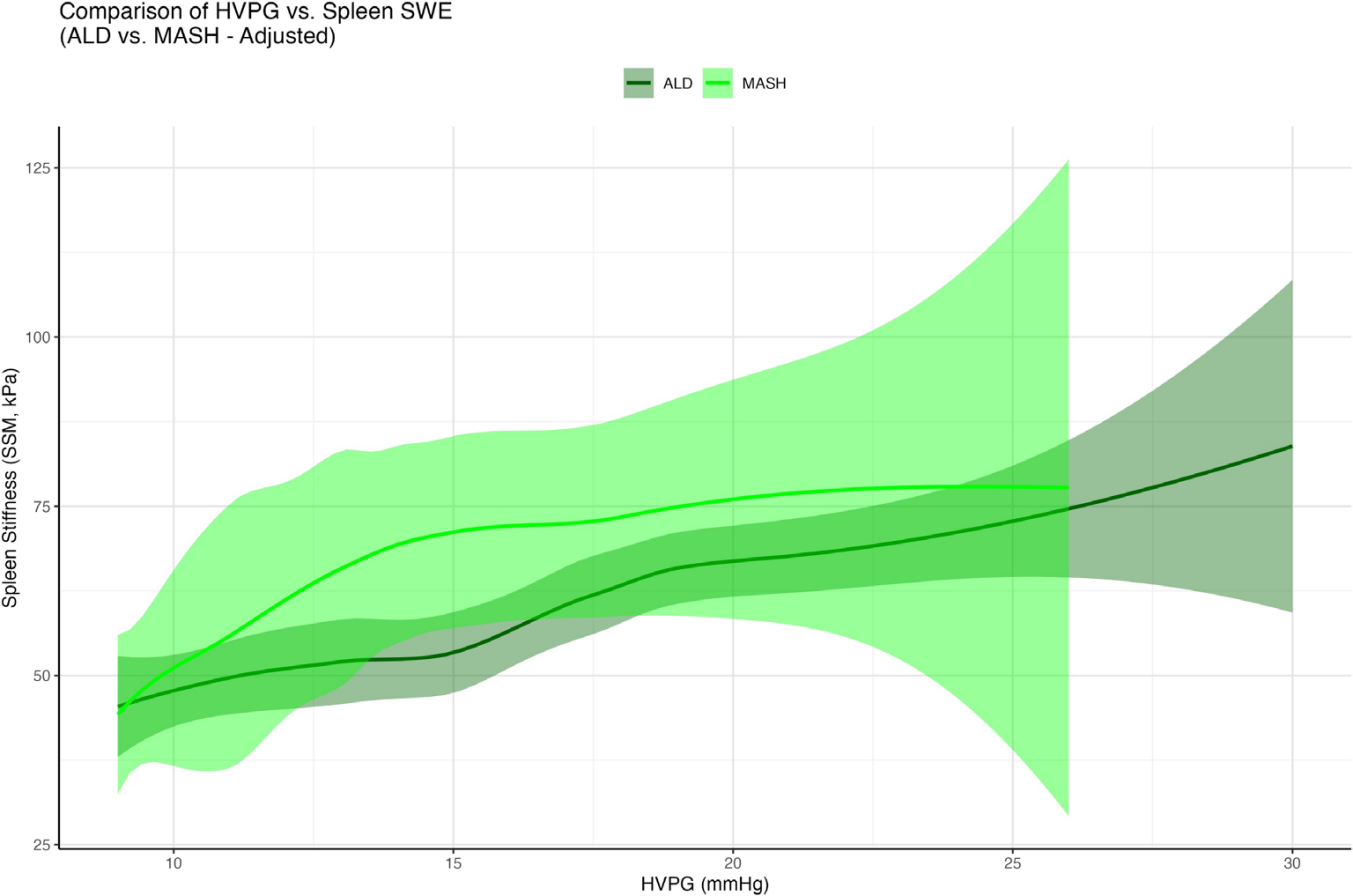
Datapoint‐based model showing SSM compared to HVPG in ALD vs. MASH. Datapoint‐based models of SSM vs. HVPG (with confidence intervals (transparent areas)) are shown for ALD (dark green) and MASH (bright green) patients separately. For modelling reasons, any HVPG value < 10 mmHg was set to 9 mmHg. Importantly, in the clinically relevant range of > 12 mmHg (i.e., when variceal bleeding can occur) at any given HVPG, the SSM was higher in MASH patients than in ALD patients—suggesting a presinusoidal PH component, not reflected by HVPG but by SSM.

Importantly, in the clinically relevant range of > 12 mmHg (i.e., when variceal bleeding can occur) at any given HVPG, the 2D‐SWE‐SSM was higher in MASH patients than in ALD patients—suggesting a presinusoidal PH component, not reflected by HVPG but by 2D‐SWE‐SSM (Figure 3). In contrast, this pattern was not observed for LSM (Figure S2).

In a multivariable linear regression model adjusting for BMI, diabetes mellitus, statin intake, and arterial hypertension, MASH remained an independent predictor of a higher SSM/LSM Ratio compared to ALD (*β* = 0.59, *p* = 0.046).

Notably, patients with an Aspartate transaminase (AST) > 75 U/L had a significantly lower 2D‐SWE‐SSM/LSM than patients < 75 U/L (AST < 75 U/L: 1.60 [1.05–2.80]; AST > 75 U/L: 1.27 [0.823–1.54]; *p* = 0.001).

### 3.4 PH Surrogates in MASH Patients With Higher 2D‐SWE‐SSM/LSM Ratio Than ALD Patients

Maximal spleen diameters as another integral surrogate of PH severity were compared across aetiologies (Table 1). The overall median spleen size was 13.3 [11.7–15.5] cm, with 69.9% of our patient cohort presenting splenomegaly (spleen diameter > 12 cm). While ALD patients had more severe liver disease indicated by MELD (ALD: 11.0 [10.0–15.3] vs. MASH: 9.00 [8.00–11.0], *p* < 0.001) and HVPG (ALD: 15.0 [11.0–19.0] mmHg vs. MASH: 12.0 [8.00–15.0] mmHg; *p* < 0.001) than MASH, the median spleen diameters were similar (or even slightly larger) in MASH (13.6 [11.5–14.8] cm) and/than in ALD (13.1 [11.7–15.0] cm). Importantly, if spleen diameters were normalised to “intrahepatic” PH severity (i.e., adjusted for HVPG), MASH patients showed significantly larger spleen diameters per mmHg‐HVPG than ALD patients (0.85 cm vs. 1.15 cm per mmHg‐HVPG; *p* < 0.001).

Varices (MASH: 34.7%, ALD: 47.0%, *p* = 0.162) and PLT (MASH: 127 [92.0–171] G/L vs. ALD: 110 [79.0–163] G/L; *p* = 0.174) were similar in MASH and in ALD patients. The proportion of patients with elevated von Willebrand factor antigen plasma levels (VWF) > 240%, a strong surrogate for CSPH, was significantly different between ALD and MASH.

Since statins are frequently prescribed to patients with MASH and may influence endothelial dysfunction as well as the severity of liver disease and PH, we compared MASH patients with and without statin use (Table S3). Notably, liver disease severity—as indicated by MELD and HVPG—was comparable between the two groups, and VWF levels did not differ between MASH patients with and without statin use (*p* = 0.624).

### 3.5 Comparing Outcomes and PH Surrogates Between Patients With High SSM/LSM vs. Low SSM/LSM Ratio

The PH surrogate markers were further analysed by splitting the cohort into tertiles based on 2D‐SWE‐SSM/LSM ratio (Table S2).

Platelet count (PLT) (Low SSM/LSM ratio: 106 [77.0–160] G/L; High SSM/LSM ratio: 122 [80.0–173] G/L; *p* = 0.433), spleen size (Low SSM/LSM ratio: 13.2 [11.7–16.0] cm; High SSM/LSM ratio: 13.5 [11.8–15.3] cm; *p* = 0.783) and VWF levels (Low SSM/LSM ratio: 264 [208–329] %; High SSM/LSM ratio: 247 [185–328] %; *p* = 0.522) showed no significant difference between SSM/LSM ratio tertiles.

Among MASH patients, those with a high SSM/LSM ratio (above the median) showed a numerically higher cumulative incidence of decompensation or liver‐related death compared to those below the median—13% vs. 5% at 6 months (*p* = 0.67) and 18% vs. 5% at 1 year (*p* = 0.67; Figure S3).

## 4 Discussion

The results of our study demonstrate significant differences in the 2D‐SWE‐SSM/LSM ratios across distinct liver disease aetiologies. Specifically, MASH patients exhibited significantly higher 2D‐SWE‐SSM/LSM ratios compared to ALD patients. Although 2D‐SWE‐SSM values were comparable between these groups, the difference in the ratios was primarily driven by significantly higher 2D‐SWE‐LSM values in ALD patients. Post hoc power analysis confirmed that the study was adequately powered to detect the observed mean difference.

Notably, MASH patients presented with lower HVPG and MELD scores than ALD patients, indicating less severe liver disease—a finding that could account for the lower 2D‐SWE‐LSM values in MASH. To control for this disparity in disease (fibrosis) severity, we normalised the 2D‐SWE‐SSM/LSM ratio for 2D‐SWE‐LSM (i.e., a valuable surrogate of the intrahepatic PH component) [33]. Even after this adjustment, the 2D‐SWE‐SSM/LSM ratio remained significantly elevated in MASH compared to ALD patients.

When controlling for key metabolic and cardiovascular risk factors, such as BMI, diabetes mellitus, statin use, and arterial hypertension, our multivariable regression analysis confirmed that MASH remained independently associated with a higher SSM/LSM ratio compared to ALD. This finding suggests that the elevated ratio in MASH is not merely a reflection of its associated comorbidities but rather stems from intrinsic pathophysiological alterations unique to the disease.

While the median 2D‐SWE‐SSM values were comparable between ALD and MASH patients, database point models revealed that MASH patients consistently demonstrated higher 2D‐SWE‐SSM values than ALD patients at equivalent HVPG. These findings suggest the presence of a presinusoidal PH component in MASH that is detected by SSM but not captured by HVPG measurements.

In accordance with prior research [29], across all aetiologies, PSVD patients had the highest 2D‐SWE‐SSM/LSM ratios in our study. It has been suggested that the 2D‐SWE‐SSM/LSM ratio is higher in aetiologies with a potential presinusoidal PH component, such as PSVD [28] or in aetiologies with portal inflammation, such as viral hepatitis C [21]. Notably, MASH patients tend to decompensate at lower HVPG levels than HCV patients [12]. Therefore, our findings support the concept of a presinusoidal component for MASH, which is not reflected by HVPG measurements but still impacts the risk of hepatic decompensation. Our results of a lower SSM/LSM ratio in ALD vs. MASH are consistent with those reported by Elshaarawy et al., who found lower SSM/LSM ratios in ALD vs. HCV [21].

To further test this hypothesis, we assessed other PH surrogates, including splenomegaly, PLT, and VWF [34] on top of 2D‐SWE‐SSM and 2D‐SWE‐SSM/LSM ratios across aetiologies. Interestingly, MASH patients—notably with less severe liver disease—and ALD patients showed similar median spleen diameter and PLT. Only VWF levels were lower compared to ALD patients. Importantly, when we adjusted for liver disease severity, and of particular relevance for ‘sinusoida’ PH severity (i.e., for HVPG), the spleen diameter was significantly higher in MASH in comparison to ALD patients. Interpreting these results, we would hypothesise that MASH patients may indeed have an (additional) presinusoidal PH component that is reflected by their 2D‐SWE‐SSM/LSM ratio. The higher VWF levels may reflect the aggravated endothelial dysfunction related to prevalent cardiovascular risk factors in MASH patients [35].

Our analysis uncovered a prognostic signal for the SSM/LSM ratio in MASH, with patients in the higher‐ratio group experiencing a higher number of liver‐related events. While this relevant observation was only a non‐significant trend, it represents the first evidence for the prognostic utility of the SSM/LSM ratio as a novel, non‐invasive biomarker in MASH patients. Larger, prospective studies are warranted to definitively establish the SSM/LSM ratio as a tool for clinical risk stratification in patients with MASH.

Previous studies have shown that HVPG correlates well with directly measured PP in liver disease causing sinusoidal PH, such as ALD [30, 36], but correlates less strongly in aetiologies with presinusoidal PH [8, 9]. The correlation between ‘indirectly‐measured’ HVPG and ‘true’ PP in sinusoidal liver diseases has been well studied in patients with cirrhosis due to ALD or viral hepatitis [30, 36]. Despite a lack of data for MASH patients [37], recently it has been suggested that MASH could have a presinusoidal component that is not fully reflected by HVPG [10, 11, 12]. Consequently, the 2D‐SWE‐SSM/LSM ratio could provide an additional assessment of PH severity.

Our study has limitations: First, the small sample size of MASH patients; however, the detailed characterisation and consecutive selection for this study seem to compensate for this. Second, the different liver disease severities in MASH and ALD patients; however, this likely reflects the clinical scenario of a more rapid course of ALD patients who have been repeatedly shown to be at higher risk for liver disease progression [38]. Importantly, we performed adjusted analyses that still supported the concept of a presinusoidal PH component in MASH patients. Third, we did not perform direct portal pressure measurements in our study; however, these methods—due to their invasive nature—are not routinely performed in clinical practice. Additionally, endosonographic‐based portal pressure gradient (EUS‐PPG) measurements, although less invasive, have inherent limitations and are also not (yet) implemented in clinical routine [18]. Given these constraints, we relied on a comprehensive assessment of PH using a combination of HVPG measurements and both 2D‐SWE‐LSM and 2D‐SWE‐SSM—obtained by trained operators using an advanced 2D‐SWE system [39]. Finally, the lack of histological data to evaluate the presinusoidal component of PH highlights the need for further research. Specifically, immunohistochemical staining for vascular and biliary markers, coupled with morphometric analysis, could provide valuable insights into functional and structural changes causing a presinusoidal PH component in MASH patients.

In conclusion, our study demonstrates that 2D‐SWE metrics vary by liver disease aetiology. Specifically, the elevated SSM/LSM ratio observed in PSVD and MASH suggests a presinusoidal component of PH—a factor not fully captured by HVPG, which is mostly reflective of the sinusoidal PH typical of ALD and viral disease. Further studies specifically designed to investigate the correlation between the higher 2D‐SWE‐SSM/LSM ratio with directly measured PPG are warranted. Additionally, it remains to be explored if the elevated 2D‐SWE‐SSM/LSM ratio in MASH is associated with clinical outcomes, i.e., liver‐related events.

## Author Contributions

All authors contributed either to study concept and design (C.S., T.R., D.J.M.B.) and/or data acquisition (all authors), analysis (C.S., T.R., D.J.M.B.) or interpretation (all authors). C.S., T.R. and D.J.M.B. drafted the manuscript, which was critically revised by all other authors. All authors read and approved the final manuscript.

## Disclosure

The authors have nothing to report.

## Ethics Statement

The local ethics committee approved the study protocol (EK‐number: 1544/2019) and informed consent was obtained from all patients.

## Conflicts of Interest

The authors declare no conflicts of interest.

## Supporting information


**Figure S1:** liv70261‐sup‐0001‐Figures.docx.
**Figure S2:** liv70261‐sup‐0001‐Figures.docx.
**Figure S3:** liv70261‐sup‐0001‐Figures.docx.

**Table S1:** liv70261‐sup‐0002‐TableS1.docx.

**Table S2:** liv70261‐sup‐0003‐TableS2.docx.

**Table S3:** liv70261‐sup‐0004‐TableS3.docx.

## Data Availability

The data that support the findings of this study are available from the corresponding author upon reasonable request.
