# Supplementary material for: Diagnostic and Clinical Implications of High Spleen‐To‐Liver Stiffness Ratio in MASH—A Prospective, Comparative Study
Source: Liver Int. 2025 Aug 30;45(10):e70261. doi: 10.1111/liv.70261 (PMC12397721; doi:10.1111/liv.70261)
Supplement: Supplementary file 1 — Figure S1: liv70261‐sup‐0001‐Figures.docx. Figure S2: liv70261‐sup‐0001‐Figures.docx. Figure S3: liv70261‐sup‐0001‐Figures.docx. [file LIV-45-0-s003.docx]

**Supplement:**

**Supplementary Figure-S1: Scatterplots of 2D-SWE-SSM/LSM ratio to HVPG in the overall group, ALD and MASH.**


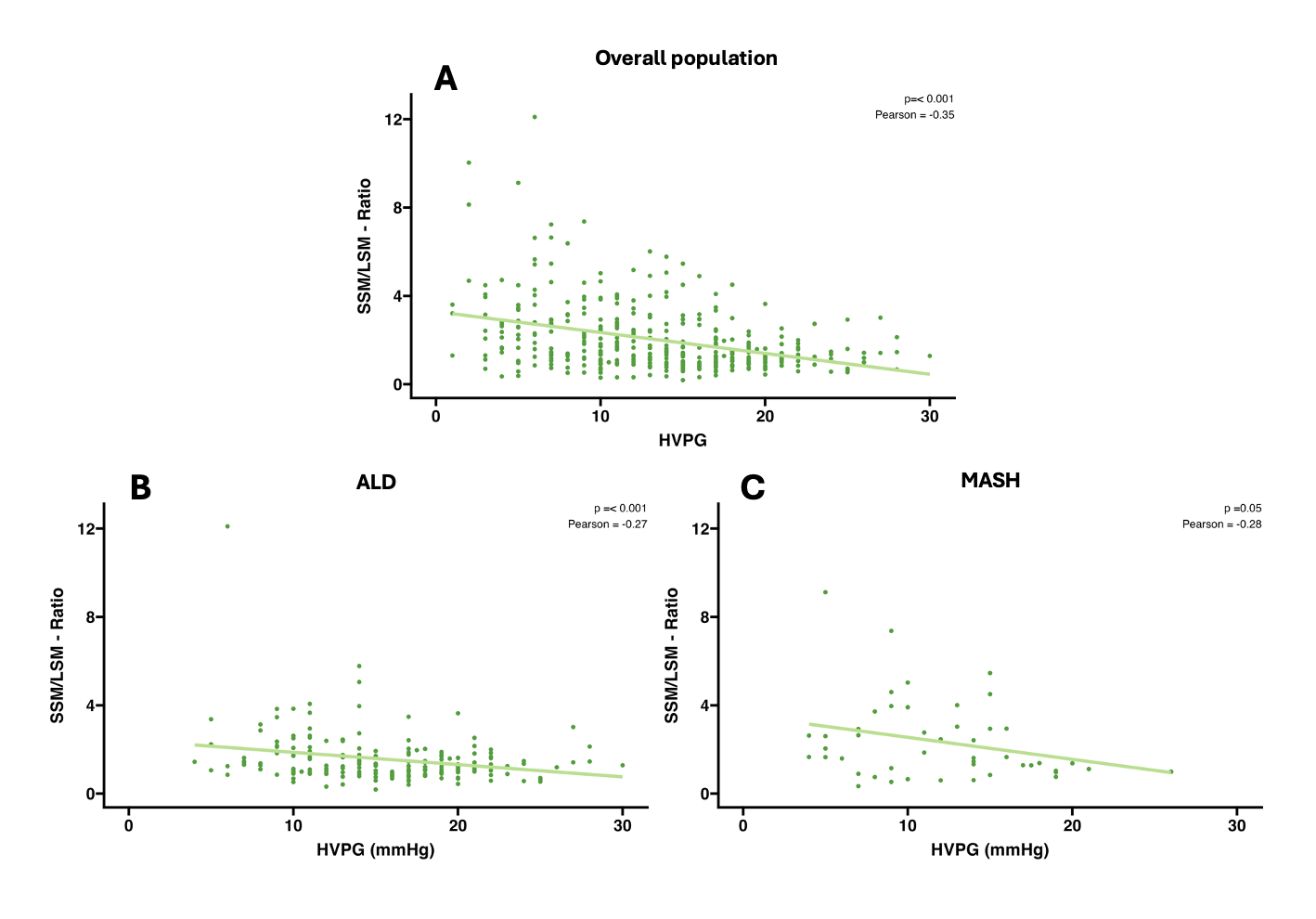


Scatterplots of SSM/LSM ratio (y-axis) to HVPG (x-axis) in the overall group (A), ALD (B) and MASH (C). Abbreviations: ALD – Alcoholic liver disease, HVPG – hepatic venous pressure gradient, LSM – liver stiffness measurement, MASH – metabolic dysfunction steatohepatitis, SSI – supersonic shear imaging, SSM – Spleen stiffness measurement

**Supplementary Figure-S2: Datapoint based model showing LSM compared to HVPG in ALD vs MASH**

**
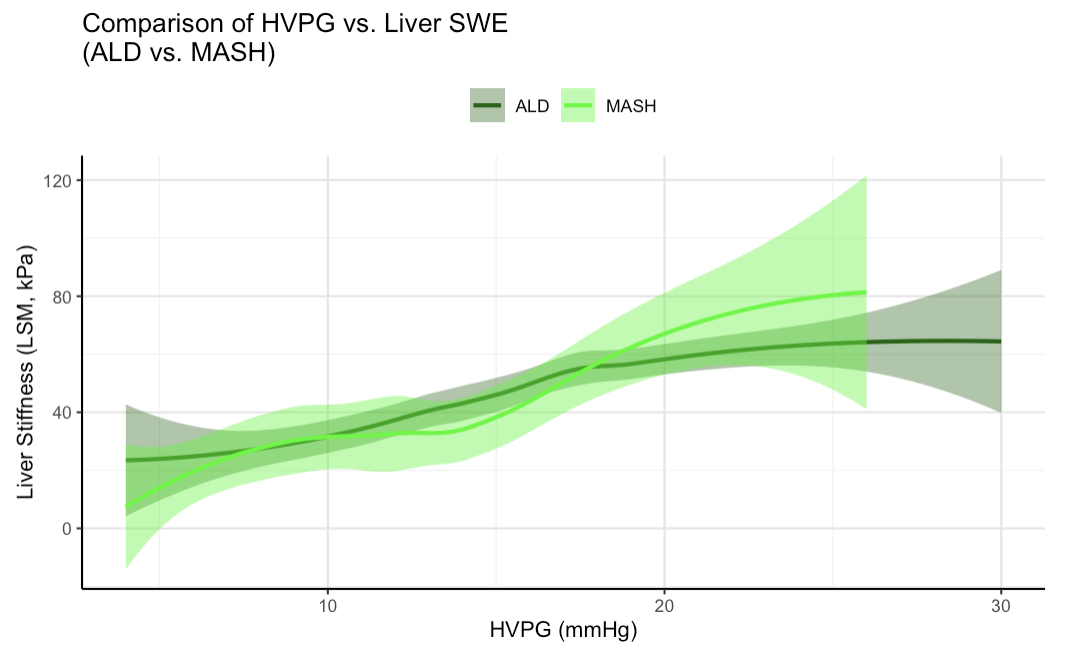
**

**Figure-Legend.** Datapoint-based models of LSM vs. HVPG (with confidence intervals (transparent areas)) are shown for ALD (dark green) and MASH (bright green) patients separately.
The modelled relationship between LSM and HVPG was very similar in ALD and MASH patients.
Abbreviations: ALD – Alcoholic liver disease, HVPG – hepatic venous pressure gradient, LSM – liver stiffness measurement, MASH – metabolic dysfunction associated steatohepatitis, SSM – Spleen stiffness measurement

**Supplementary Figure S3: Cumulative Incidence of first decompensation or liver related death with LT, HCC and non-liver related death as competing risks**


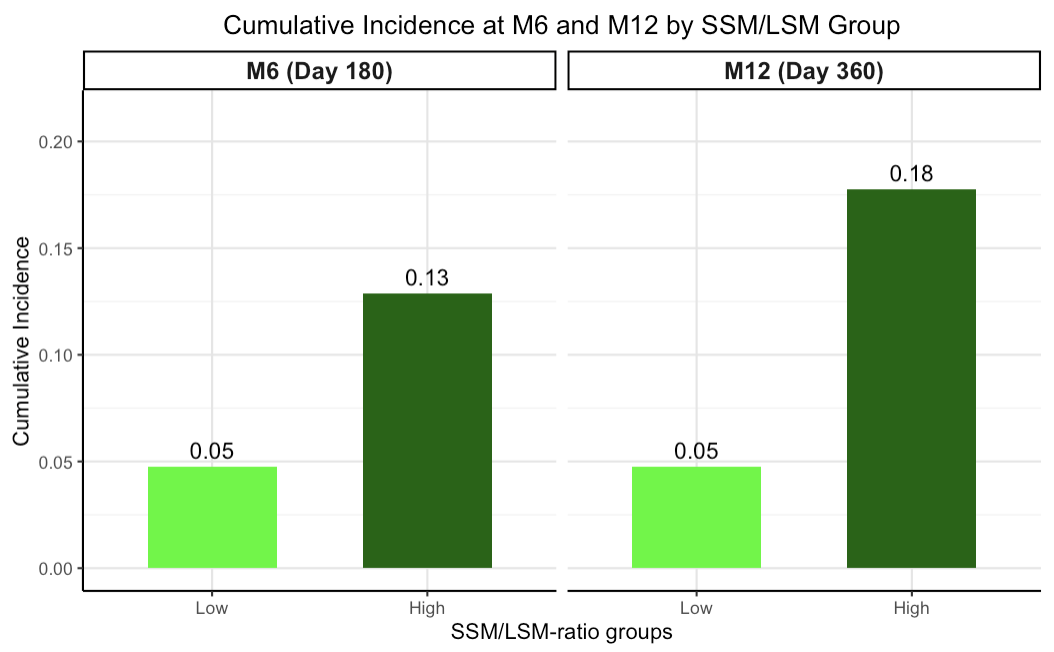


**Figure Legend:** Cumulative incidence (%) at Months 6 and 12, stratified by SSM/LSM ratio groups.

Abbreviations: LSM – liver stiffness measurement, SSM – spleen stiffness measurement

**Supplementary methods:**

The following R packages were used: base 1.4.1, bit 4.0.5, caret 6.0-94, datasets 4.3.2, DBI 1.2.0, DiagrammeR 1.0.11, dplyr 1.1.4, ff 4.0.12, flextable 0.9.5, forcats 1.0.0, ggplot2 3.4.4, ggprism 1.0.4, glue 1.7.0, Gmisc 3.0.3, graphics 4.3.2, grDevices 4.3.2, grid 4.3.2, gtsummary 1.7.2, hgutils 0.2.11, htmlTable 2.4.2, knitr 1.45, lattice 0.21-9, lubridate 1.9.3, magrittr 2.0.3, methods 4.3.2, pacman 0.5.1, purr 1.0.2, RColorBrewer 1.1-3, Rcpp 1.0.12, readr 2.1.5, readxl 1.4.3, RecordLinkage 0.4-12.4, reshape2 1.4.4, RSQLite 2.3.5, stats 4.3.2, stringdist 0.9.12, stringr 1.5.1, table1 1.4.3, tibble 3.2.1, tidyr 1.3.1, tidyverse 2.0.0, utils 4.3.2, yardstick 1.3.1.
