# Supplementary material for: Diagnostic and Clinical Implications of High Spleen‐To‐Liver Stiffness Ratio in MASH—A Prospective, Comparative Study
Source: Liver Int. 2025 Aug 30;45(10):e70261. doi: 10.1111/liv.70261 (PMC12397721; doi:10.1111/liv.70261)
Supplement: Supplementary file 2 — Table S1: liv70261‐sup‐0002‐TableS1.docx. [file LIV-45-0-s002.docx]

**Supplementary Table-S1: Patient characteristics for all different etiologies.**

|  | **Total Population** | **Viral** | **ALD** | **MASH** | **AIH and Cholestatic** | **PSVD** | **Other/ Cryptogenic** | **P-value** |
| --- | --- | --- | --- | --- | --- | --- | --- | --- |
|  | **N=399** | **N=59** | **N=200** | **N=49** | **N=40** | **N=18** | **N=33** | **(without overall)** |
| **Age (years)** | 56.0 [48.5 - 64.0] | 54.0 [48.5 - 64.0] | 57.0 [51.0 - 63.3] | 61.0 [56.0 - 65.0] | 54.5 [31.8 - 61.3] | 51.0 [41.8 - 55.8] | 48.0 [34.0 - 64.0] | 0.001 |
| **Male sex (n, %)** | 128 (32.1%) | 41 (69.5%) | 52 (26.0%) | 19 (38.8%) | 20 (50.0%) | 11 (61.1%) | 21 (63.6%) | 0.050 |
| **BMI** | 26.0 [22.5 - 30.0] | 25.8 [22.4 - 29.2] | 26.0 [22.7 - 29.9] | 32.8 [27.9 - 36.8] | 22.3 [21.0 - 27.2] | 26.4 [22.7 - 29.4] | 25.9 [21.9 - 26.6] | < 0.001 |
| **HVPG** | 14.0 [9.00 - 18.0] | 11.0 [7.00 - 16.5] | 15.0 [11.0 - 19.0] | 12.0 [8.00 - 15.0] | 13.5 [9.00 - 17.0] | 5.50 [4.25 - 7.00] | 10.0 [4.00 - 17.0] | < 0.001 |
| **CSPH** | 289 (72.4%) | 38 (64.4%) | 173 (86.5%) | 31 (63.3%) | 29 (72.5%) | 1 (5.6%) | 17 (51.5%) | < 0.001 |
| **VCTE-LSM (kPa)** | 31.3 [16.8 - 54.2] | 21.1 [11.6 - 32.2] | 42.9 [26.3 - 65.6] | 24.4 [16.9 - 43.1] | 23.8 [16.3 - 41.1] | 7.30 [6.08 - 11.3] | 22.0 [6.95 - 46.1] | < 0.001 |
| **2D-SWE-LSM (kPa)** | 33.2 [16.5 - 59.8] | 23.5 [13.8 - 41.9] | 45.5 [25.1 - 63.9] | 20.9 [15.7 - 55.0] | 22.2 [13.9 - 38.3] | 11.2 [7.85 - 14.4] | 15.9 [9.60 - 45.7] | < 0.001 |
| **2D-SWE-SSM (kPa)** | 53.9 [35.0 - 69.3] | 53.0 [36.7 - 68.4] | 58.8 [39.7 - 71.4] | 52.8 [34.3 - 82.6] | 48.3 [36.8 - 54.2] | 42.6 [33.3 - 61.9] | 50.2 [23.6 - 62.9] | 0.009 |
| **2D-SWE-SSM/LSM ratio** | 1.48 [1.00 - 2.62] | 2.10 [1.24 - 3.05] | 1.28 [0.963 - 1.83] | 1.66 [1.22 - 2.94] | 1.65 [1.19 - 3.26] | 3.19 [1.96 - 5.45] | 2.24 [1.04 - 3.13] | < 0.001 |
| **2D-SWE-SSM/LSM ratio/LSM** | 0.0468 [0.0174 - 0.163] | 0.0828 [0.0289 - 0.210] | 0.0290 [0.0155 - 0.0669] | 0.0958 [0.0197 - 0.202] | 0.0829 [0.0275 - 0.253] | 0.247 [0.171 - 0.662] | 0.111 [0.0246 - 0.287] | < 0.001 |
| **MELD** | 11.0 [9.00 - 14.0] | 10.0 [8.00 - 12.0] | 11.0 [10.0 - 15.3] | 9.00 [8.00 - 11.0] | 12.0 [8.00 - 15.0] | 9.00 [7.00 - 12.8] | 11.5 [8.25 - 14.0] | < 0.001 |
| **Platelets (G/L)** | 110 [77.0 - 165] | 94.0 [60.0 - 134] | 110 [79.0 - 163] | 127 [92.0 - 171] | 102 [71.0 - 144] | 87.0 [53.0 - 157] | 135 [89.0 - 179] | 0.051 |
| **Thrombocytopenia <150 G/L (n, %)** | 253 (63.4%) | 44 (74.6%) | 119 (59.5%) | 31 (63.3%) | 29 (72.5%) | 13 (72.2%) | 17 (51.5%) | < 0.001 |
| **AST (U/L)** | 39.0 [29.0 - 58.0] | 36.0 [25.0 - 46.0] | 38.0 [28.0 - 58.0] | 43.5 [33.3 - 58.8] | 51.5 [37.0 - 112] | 32.0 [24.0 - 41.0] | 40.0 [28.0 - 57.0] | < 0.001 |
| **ALT (U/L)** | 30.0 [22.0 - 47.0] | 31.0 [23.0 - 45.0] | 27.0 [20.0 - 36.0] | 43.5 [29.0 - 67.0] | 55.0 [30.3 - 97.0] | 32.5 [22.3 - 50.0] | 30.0 [21.0 - 47.5] | < 0.001 |
| **GGT (U/L)** | 86.0 [40.0 - 169] | 39.0 [27.0 - 56.0] | 107 [56.0 - 214] | 114 [62.5 - 208] | 112 [54.8 - 227] | 66.0 [29.3 - 117] | 98.0 [31.5 - 180] | < 0.001 |
| **vWF (%)** | 261 [193 - 331] | 228 [175 - 321] | 283 [216 - 366] | 232 [184 - 315] | 270 [233 - 301] | 180 [141 - 241] | 212 [152 - 308] | < 0.001 |
| **Varices** |  |  |  |  |  |  |  |  |
| No | 172 (48.3%) | 30 (55.6%) | 83 (46.4%) | 24 (57.1%) | 10 (29.4%) | 8 (47.1%) | 17 (56.7%) | 0.018 |
| Small | 97 (27.2%) | 12 (22.2%) | 54 (30.2%) | 10 (23.8%) | 12 (35.3%) | 4 (23.5%) | 5 (16.7%) |  |
| Large | 76 (21.3%) | 9 (16.7%) | 40 (22.3%) | 7 (16.7%) | 11 (32.4%) | 5 (29.4%) | 4 (13.3%) |  |
| **Spleen (cm)** | 13.3 [11.7 - 15.5] | 13.6 [11.9 - 15.8] | 13.1 [11.7 - 15.0] | 13.6 [11.5 - 14.8] | 14.5 [12.3 - 16.9] | 15.7 [12.0 - 17.6] | 11.9 [10.3 - 16.7] | 0.059 |
| **Splenomegaly** | 279 (100%) | 44 (100%) | 138 (100%) | 35 (100%) | 33 (100%) | 13 (100%) | 16 (100%) | 0.051 |
| **(> 12cm; n, %)** |  |  |  |  |  |  |  |  |

Abbreviations: AIH – autoimmune liver disease, ALD – alcoholic liver disease, BMI – body mass index, HVPG – hepatic venous pressure gradient, LSM – liver stiffness measurement, MASH – metabolic dysfunction associated steatohepatitis, MELD – model of endstage liver disease, N – number, CSPH – clinically significant portal hypertension, PSVD – portal sinusoidal vascular disease, SSI – super sonic imaging, SSM – spleen stiffness measurement, VCTE – vibration-controlled transient elastography, vWF – von Willebrand factor, US- ultrasound.
