# Supplementary material for: Diagnostic and Clinical Implications of High Spleen‐To‐Liver Stiffness Ratio in MASH—A Prospective, Comparative Study
Source: Liver Int. 2025 Aug 30;45(10):e70261. doi: 10.1111/liv.70261 (PMC12397721; doi:10.1111/liv.70261)
Supplement: Supplementary file 3 — Table S2: liv70261‐sup‐0003‐TableS2.docx. [file LIV-45-0-s001.docx]

**Supplementary Table-S2: Patient characteristics by 2D-SWE-SSM/LSM ratio tertiles.**

|  |  | **Low 2D-SWE-SSM-LSM ratio:** | **Middle 2D-SWE-SSM-LSM ratio:** | **High 2D-SWE-SSM-LSM ratio:** | **P-value** |
| --- | --- | --- | --- | --- | --- |
|  | **Overall** |  |  |  |  |
|  | **(N=399)** | **(N=135)** | **(N=134)** | **(N=130)** |  |
| **Age (years)** | 56.0 [48.5 - 64.0] | 55.0 [47.5 - 63.0] | 59.0 [51.0 - 65.0] | 56.0 [48.0 - 63.0] | 0.096 |
| **Male sex (n, %)** | 128 (32.1%) | 86 (63.7%) | 97 (72.4%) | 88 (67.7%) | 0.312 |
| **BMI** | 26.0 [22.5 - 30.0] | 26.1 [22.2 - 29.9] | 26.4 [22.7 - 30.4] | 25.2 [22.5 - 29.9] | 0.340 |
| **HVPG** | 14.0 [9.00 - 18.0] | 14.0 [10.0 - 19.0] | 14.0 [10.0 - 18.0] | 13.0 [8.00 - 17.0] | 0.193 |
| **CSPH** | 289 (72.4%) | 104 (77.0%) | 101 (75.4%) | 84 (64.6%) | 0.066 |
| **VCTE-LSM (kPa)** | 31.3 [16.8 - 54.2] | 36.1 [18.0 - 61.1] | 29.5 [17.4 - 51.8] | 25.5 [14.5 - 48.2] | 0.056 |
| **VCTE-LSM > 25 kPa** | 221 (55.4%) | 85 (63.0%) | 72 (53.7%) | 64 (49.2%) | 0.218 |
| **2D-SWE-LSM (kPa)** | 33.2 [16.5 - 59.8] | 38.9 [17.8 - 65.6] | 33.3 [16.9 - 56.2] | 26.9 [14.7 - 54.9] | 0.041 |
| **2D-SWE-LSM > 21 kPa** | 140 (35.1%) | 39 (28.9%) | 46 (34.3%) | 55 (42.3%) | 0.252 |
| **2D-SWE-SSM (kPa)** | 53.9 [35.0 - 69.3] | 50.9 [34.9 - 67.1] | 54.0 [38.7 - 68.8] | 56.7 [34.6 - 76.3] | 0.446 |
| **2D-SWE-SSM > 40 kPa** | 271 (67.9%) | 89 (65.9%) | 97 (72.4%) | 85 (65.4%) | 0.661 |
| **2D-SWE-SSM/LSM ratio** | 1.48 [1.00 - 2.62] | 1.16 [0.876 - 2.22] | 1.60 [1.21 - 2.38] | 1.87 [1.18 - 3.06] | < 0.001 |
| **MELD** | 11.0 [9.00 - 14.0] | 11.0 [9.00 - 15.0] | 11.0 [9.00 - 14.0] | 11.0 [8.00 - 14.0] | 0.396 |
| **Platelet (G/L)** | 110 [77.0 - 165] | 106 [77.0 - 160] | 110 [73.3 - 156] | 122 [80.0 - 173] | 0.433 |
| **Thrombocytopenia <150 G/L (n, %)** | 253 (63.4%) | 91 (67.4%) | 86 (64.2%) | 76 (58.5%) | 0.501 |
| **vWF (%)** | 261 [193 - 331] | 264 [208 - 329] | 260 [197 - 336] | 247 [185 - 328] | 0.522 |
| **NH3 (µmol/L)** | 30.4 [23.3 - 44.1] | 34.1 [24.8 - 43.9] | 31.4 [23.9 - 45.3] | 27.3 [23.0 - 41.7] | 0.254 |
| **ALP (mU/mL)** | 98.0 [75.0 - 134] | 95.0 [76.0 - 136] | 105 [79.5 - 141] | 93.0 [72.0 - 125] | 0.245 |
| **CYFRA 21** | 2.80 [2.00 - 4.00] | 2.90 [2.20 - 3.80] | 2.70 [1.90 - 4.15] | 2.80 [1.90 - 4.00] | 0.905 |
| **Varices (n, %)** | 168 (43.3%) | 61 (45.2%) | 54 (41.3%) | 58 (44.6%) | 0.557 |
| **Spleen (cm)** | 13.3 [11.7 - 15.5] | 13.2 [11.7 - 16.0] | 13.1 [11.6 - 15.5] | 13.5 [11.8 - 15.3] | 0.783 |
| **Splenomegaly > 12 cm (n, %)** | 279 (69.9%) | 92 (68.1%) | 91 (67.9%) | 96 (73.8%) | 0.494 |

Abbreviations: BMI – Body Mass Index, HVPG – Hepatic Venous Pressure Gradient, LSM – Liver Stiffness Measurement, MELD – Model of Endstage Liver Disease, N – Number, CSPH – Clinically Significant Portal Hypertension, SSI – Super Sonic Imaging, SSM – Spleen Stiffness Measurement, vWF – von Willebrand factor, US- Ultrasound.
