# Supplementary material for: Diagnostic and Clinical Implications of High Spleen‐To‐Liver Stiffness Ratio in MASH—A Prospective, Comparative Study
Source: Liver Int. 2025 Aug 30;45(10):e70261. doi: 10.1111/liv.70261 (PMC12397721; doi:10.1111/liv.70261)
Supplement: Supplementary file 4 — Table S3: liv70261‐sup‐0004‐TableS3.docx. [file LIV-45-0-s004.docx]

**Supplementary Table- S3: Comparison of MASH patients with and without statin use**

| **MASH** |  |  |  |
| --- | --- | --- | --- |
|  | **No Statin** | **Statin** | **P-value** |
|  | **(N=24)** | **(N=25)** |  |
| **Age (years)** | 58.0 [51.0 - 63.3] | 63.0 [57.0 - 68.0] | 0.0329 |
| **Male (N)** | 14 (58.3%) | 16 (64.0%) | 0.827 |
| **BMI** | 34.2 [27.1 - 36.7] | 32.2 [28.9 - 37.0] | 0.904 |
| **HVPG (mmHg)** | 10.5 [7.00 - 16.0] | 13.0 [9.00 - 15.0] | 0.521 |
| **CSPH (N)** | 14 (58.3%) | 17 (68.0%) | 0.195 |
| **VCTE-LSM (kPa)** | 25.3 [16.6 - 44.6] | 24.4 [18.7 - 36.8] | 0.861 |
| **MELD** | 9.00 [8.00 - 11.0] | 9.00 [8.00 - 11.0] | 0.648 |
| **vWF (%)** | 242 [198 - 303] | 217 [164 - 322] | 0.624 |
| **Child Pugh Score** | 7.00 [5.75 - 8.00] | 6.00 [5.00 - 8.00] | 0.365 |

Abbreviations: BMI – Body Mass Index, HVPG – Hepatic Venous Pressure Gradient, LSM – Liver Stiffness Measurement, MELD – Model of Endstage Liver Disease, N – Number, CSPH – Clinically Significant Portal Hypertension, SSI – Super Sonic Imaging, vWF – von Willebrand factor, US- Ultrasound.
